# Hemosiderotic Dermatofibroma: A Rare and Atypical Variant Capable of Clinically Resembling Melanoma

**DOI:** 10.7759/cureus.6736

**Published:** 2020-01-22

**Authors:** Tomer Lagziel, Scott Sylvester, Charles S Hultman, Mohammed Asif

**Affiliations:** 1 Plastic Surgery, The Johns Hopkins University School of Medicine, Baltimore, USA

**Keywords:** dermatofibroma, melanoma, hemosiderotic, skin lesion

## Abstract

A dermatofibroma (DF) is a common, benign tumor composed of fibroblastic and histiocytic cells. DF presents clinically with several different reported variants. One rare variant is hemosiderotic DF (HDF), which is made up of small blood vessels and hemosiderin deposits. HDF can be indistinguishable, clinically, from melanoma, making the use of other pathological tools crucial in the diagnosis.

We report the case of a 25-year-old male medical student from the Caribbean who presented to our clinic with a single asymptomatic pigmented cystic lesion on his left posterior calf. The cystic lesion was excised surgically. Histopathology examination of the excised mass revealed a moderately cellular, poorly demarcated, dermal, fibrohistiocytic proliferation. Pathology consultation confirmed a diagnosis of HDF.

## Introduction

A dermatofibroma (DF) is a common, benign tumor composed of fibroblastic and histiocytic cells [1]. DFs present clinically with several different reported variants, accounting for approximately 3% of all skin lesion specimens received by dermatopathologists [2]. The classic presentation of DF is on a limb as a small, raised, cutaneous nodule, reddish-brown in color. One particularly rare variant of the skin condition is hemosiderotic DF (HDF), which is composed of multiple small vessels, extravasated erythrocytes, siderophages, and hemosiderin deposits [3]. Given its typical asymmetrical borders and color variation, HDF cannot be clinically differentiated from melanoma [4]. Melanoma is now regarded as the fifth most common cancer in men and the sixth most common cancer in women in the United States [5]. Wide local excision (WLE) with negative margins remains the gold standard for treating suspected melanoma lesions [5]. We report a case where a suspicious lesion was excised from a young male and histopathological examination confirmed HDF. To the best of our knowledge, no other cases of HDF have been reported in the United States.

## Case presentation

**Figure 1:**
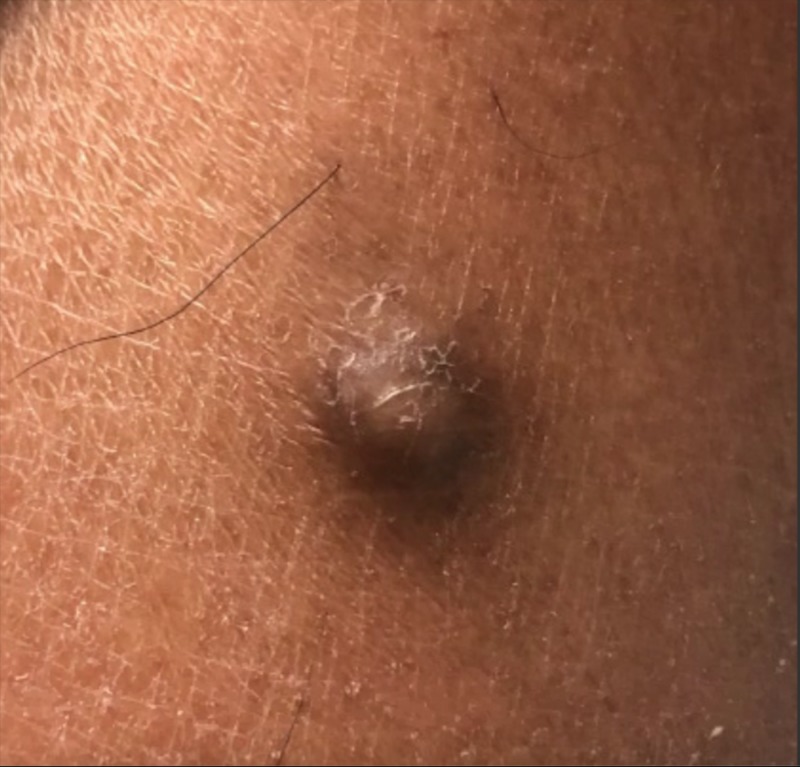
Photograph of cystic lesion, as seen during the latest consultation.

We report the case of a 25-year-old male medical student from the Caribbean who presented to our clinic with a single asymptomatic pigmented cystic lesion on his left posterior calf (Figure 1). The lesion was tracked and recorded for approximately two years and has been gradually growing in size. The mass has never been drained and has never been infected. 

The patient is otherwise healthy and has reported no fevers, chills, nausea, or vomiting. As medical student in Antigua with decreased access to medical care, he decided to seek surgical intervention due to recent mild discomfort.

Further clinical examination confirmed a circumference of 1 cm for the cystic lesion. Physical exam revealed no fluctuance, pericystic erythema, or any tenderness to palpation. Excision was planned for one week after the initial consultation.

The procedure was performed in an outpatient setting under local anesthesia. The patient was injected with 4 ml of local 1% lidocaine with epinephrine subcutaneously surrounding the 1 cm lesion. An elliptical incision was marked and carried out to excise the cystic lesion in a single specimen piece. The entire cystic lesion was subsequently sent for pathological analysis. The patient tolerated the procedure well.

The pathology report confirmed an excision of tan-brown skin lesion, measuring 1.0 x 0.8 x 0.7 cm. The cutaneous surface was remarkable for a central 0.8 x 0.6 cm gray-tan, slightly raised, firm nodule. 

**Figure 2:**
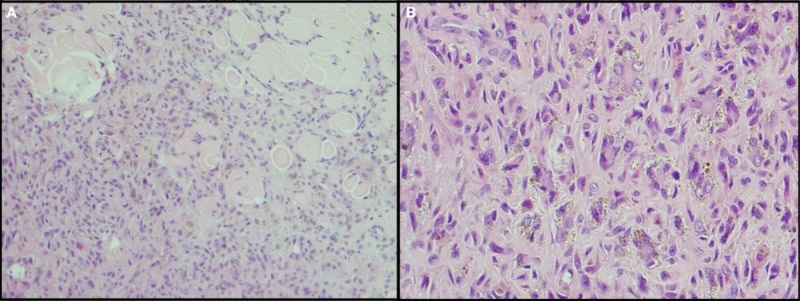
Hemosiderotic fibrous histiocytoma. (A) Intradermal cellular proliferation of spindle to fusiform fibrohistiocytic cells (H&E, 40x). (B) Scattered hemosiderin-laden multinucleated giant cells (H&E, 100x).

Histopathological examination of the excised cystic lesion revealed a moderately cellular, poorly demarcated, dermal, fibrohistiocytic proliferation made primarily of fusiform to stellate cells with unvarying, oval nuclei and amphiphilic cytoplasm (Figure 2A), associated with intralesional pools of extravasated blood and peripheral collagen trapping. Multinucleated giant cells with peripheral cytoplasmic deposits of hemosiderin were also observed in a scattered formation (Figure 2B). A diagnosis of HDF was confirmed.

## Discussion

DFs are relatively common skin lesions, approximately 3% of all skin lesions, appearing most often in the lower extremities of young adult females [1,6]. Atypical variants of DF, such as HDF (once progressed to aneurysmal DF [ADF]), are more likely to have high proliferative activity and recurring metastasis with metastasis noted as early as three months after first diagnosis [7,8]. In contrast, the median time-to-metastasis for localized melanoma is 28 months [9]. The hemosiderin deposits and dermal blood-filled cavities, characteristic of HDF, histopathologically resemble melanoma or vascular tumors [1,10]. Concordant with our report, studies published by Han et al. and Ferrari et al. also found significant association between melanoma-like HDF lesions, especifically in males [2,11]. Although DF is usually easily identified and diagnosed clinically or dermoscopically, atypical variants make it more difficult to diagnose especially when resembling melanoma. The physical presentation of HDF and melanoma is similar and can appear as a variably pigmented papule, plaque, or hardened nodule [12]. In cases where the HDF is not removed, it is hypothesized that the red blood cells would extravasate from small vessels, eventually producing hemosiderin, which would be taken up by the tumor cells [13]. Over time, the extravasation would create intravascular spaces, thereby increasing internal tumor pressure and creating endothelial damage [13]. At this stage, the HDF could be considered an early ADF [14]. Due to the potentially aggressive nature of melanoma, we recommend a WLE with negative margins, the current gold standard, be performed followed by an expert histopathological examination to definitively diagnose the nature of the cystic lesion [15]. A definitive diagnosis is crucial to avoid to any lethal complications, and this diagnostic method should be carried out when any suspicion presents.

## Conclusions

Melanoma is a cancer with malignant potential, and any suspicious lesion should be treated with WLE with negative margins. However, HDF is a rare tumor that looks identical to melanoma in the clinical setting. Therefore, HDF should always be included as part of the differential diagnosis, especially when the patient is a young adult and the lesion appears on a limb. Dermatoscopy and clinical evaluation are insufficient in confirming HDF or melanoma due to their similar nature, and expert histopathological examination should be performed to achieve a final diagnosis.

## References

[1] Laureano A, Fernandes C, Cardoso J (2015). Hemosiderotic dermatofibroma: clinical and dermoscopic presentation mimicking melanoma. J Dermatol Case Rep.

[2] Han TY, Chang HS, Lee JH, Lee WM, Son SJ (2011). A clinical and histopathological study of 122 cases of dermatofibroma (benign fibrous histiocytoma). Ann Dermatol.

[3] Roldan-Marin R, Barreiro-Capurro A, Garcia-Herrera A (2014). Green colour as a novel dermoscopic finding in the diagnosis of haemosiderotic dermatofibroma. Australas J Dermatol.

[4] Blum A, Jaworski S, Metzler G, Bauer J (2004). Lessons on dermoscopy: dermoscopic pattern of hemosiderotic dermatofibroma. Dermatol Surg.

[5] Rastrelli M, Tropea S, Rossi CR, Alaibac M (2014). Melanoma: Epidemiology, risk factors, pathogenesis, diagnosis and classification. In Vivo.

[6] Alves JV, Matos DM, Barreiros HF, Bartolo EA (2014). Variants of dermatofibroma: a histopathological study. An Bras Dermatol.

[7] Mentzel T, Wiesner T, Cerroni L (2013). Malignant dermatofibroma: clinicopathological, immunohistochemical, and molecular analysis of seven cases. Mod Pathol.

[8] Acar EM, Tad M, Kilitci A, Kemeriz F (2018). Hemosiderotic dermatofibroma mimicking melanoma in a 12-year-old boy: a case report. Clin Case Rep.

[9] Kelati A, Aqil N, Baybay H, Gallouj S, Mernissi FZ (2017). Beyond classic dermoscopic patterns of dermatofibromas: a prospective research study. J Med Case Rep.

[10] Faruk T (2012). Metastatic behavior in melanoma: timing, pattern, survival, and influencing factors. J Oncol.

[11] Ferrari A, Argenziano G, Buccini P (2013). Typical and atypical dermoscopic presentations of dermatofibroma. J Eur Acad Dermatol Venereol.

[12] Zaballos P, Llambrich A, Ara M, Olazarán Z, Malvehy J, Puig S (2006). Dermoscopic findings of haemosiderotic and aneurysmal dermatofibroma: report of six patients. Br J Dermatol.

[13] Villarreal DJ, Luz AT, Buçard AM, de Abreu L, Cuzzi T (2017). Hemosiderotic dermatofibroma. An Bras Dermatol.

[14] Santa Cruz DJ, Kyriakos M (1981). Aneurysmal ("angiomatoid") fibrous histiocytoma of the skin. Cancer.

[15] Pavri SN, Clune J, Ariyan S, Narayan D (2016). Malignant melanoma: beyond the basics. Plast Reconstr Surg.

